# Quality of Life (QoL) assessment in a cohort of patients with Phenylketonuria

**DOI:** 10.1186/1471-2458-14-1243

**Published:** 2014-12-04

**Authors:** Chiara Cazzorla, Luca Cegolon, Alessandro P Burlina, Andrea Celato, Pamela Massa, Laura Giordano, Giulia Polo, Aurora Daniele, Francesco Salvatore, Alberto B Burlina

**Affiliations:** Division of Inborn Metabolic Diseases, Department of Pediatrics, Padua University Hospital, Padua, Italy; Department of Cardiac, Thoracic and Vascular Sciences, Padua University Hospital, Padua, Italy; Department of Infectious Disease Epidemiology, London School of Hygiene & Tropical Medicine, London, UK; Neurological Unit, St. Bassiano Hospital, Bassano del Grappa, Italy; Department of Environmental Sciences, Biological and Pharmaceutical Technologies, Second University of Naples, Caserta, Italy; CEINGE-Biotecnologie Avanzate S.c.a.r.l., Naples, Italy; IRCCS SDN-Foundation, Naples, Italy

**Keywords:** Phenylketonuria, Tetrahydrobiopterin, Quality of life, Word Health Organization Quality Of Life questionnaire-100

## Abstract

**Background:**

Phenylketonuria (PKU) is a chronic inborn error of amino acid metabolism that requires lifelong follow-up and intervention, which may represent strains on Quality of Life (QoL). This observational study evaluated QoL in a cohort of PKU patients, using updated and detailed instruments.

**Methods:**

22 patients with mild PKU respondent to BH_4_ and 21 patients with classical PKU treated with diet were recruited in this study. Adult patients completed WHOQOL questionnaire-100 (WHOQOL-100) and pediatric patients the Pediatric QoL inventory (PedsQL^TM^). Psychiatric and mood disorders were also evaluated using TAD or BDI and STAI-Y inventories. A multivariable linear regression model was fitted to investigate the predictors of QoL, including age, sex, treatment type, length of current treatment, educational level and employment status (only for adults) as covariates. Results were presented as regression coefficients with 95% confidence interval.

**Results:**

Global QoL scores were within normal range both in patients with mild and classical disease but global QoL scores were significantly higher in patients with mild PKU under BH_4_ treatment as compared to those affected by classical disease who were under diet regimen. Furthermore, QoL significantly increased in long treated PKU patients. Among adult patients, QoL scores were significantly lower in males, in patients with lower education and in those employed or unemployed as compared to students (baseline).

**Conclusions:**

Both diet and medical treatment based upon BH_4_ seem to be associated with higher QoL in the long run. However, patients with mild PKU can rely on BH_4_ to achieve a higher Phe tolerance and a better compliance to therapy due to diet relaxation/avoidance. Some specific categories of patients with a lower QoL should be investigated more in depth, engaging with those at risk of lower treatment compliance. The questionnaires employed in the present study seemed to be able to effectively detect criticalities in QoL assessment and represent an advance from previous inventories employed in the past.

## Background

Phenylketonuria (PKU, OMIM 261600) is a complex autosomal recessive metabolic disorder caused by mutations of the gene encoding for phenylalanine hydroxylase enzyme (PAH, EC 1.14.16.1). PAH deficiency leads to an accumulation of phenylalanine (Phe) in blood and brain that gradually impairs metabolic functioning and cognitive development [1]. PAH is mainly active in the liver and requires 6R-tetrahydrobiopterin (BH_4_) as a critical cofactor. An early dietary intervention based on a Phe restricted diet and the supplementation of an amino acids mixture is able to keep Phe blood levels low, avoiding mental retardation or neurological abnormalities that have been described in untreated patients [2]. Diet should be maintained lifelong and this requires a lot of effort, especially in adulthood, owing to the growing instances of social relationships [3–5]. Indeed, PKU patients must go through frequent biochemical controls, several dietary assessments and adjustments that represent stressors for both adolescent and adult patients [6, 7]. Furthermore, in young adults, the strict adherence to diet may lead to some difficulties in building up good social relationships [8], often resulting in poor adherence to dietary regimen with risks of cognitive, neuropsychological and behavioural impairment [9, 10]. BH_4_ has been introduced since 1999 as a therapeutic option alternative to diet to manage PKU, thus widely changing the matter [7, 11]. In PKU responsive patients, BH_4_ indeed allows to maintain a significantly low level of blood Phe, despite remarkable relaxation of dietary regimen [12, 13]. Current guidelines suggest that a trial with sapropterin can be provided to all PKU patients [13], nevertheless patients with milder forms of the disease are more likely to benefit from BH_4_ treatment than classical ones.

In recent years there has been increasing attention on the effect of BH_4_ on the Quality of Life (QoL) of PKU patients previously receiving dietary intervention [14–16]. However QoL scores of PKU patients and their parents measured by current inventories are reported to be generally similar to healthy controls’ [14, 17, 18], probably as the questionnaires are not able to outline differences or criticalities expected in a chronic disease. Demirdas et al. and Ziesch et al. [14, 16], for instance, investigated QoL of PKU patients using several instruments, before and after the introduction of BH_4_ [19]. Their results showed that:

- QoL global scores were comparable to the ones of general population;
- QoL scores did not improve after BH_4_ administration and consequential relaxation of diet.

The authors attributed their findings to several reasons:

- global QoL scores reflect a true picture;
- results are the consequence of a hidden disability hard to capture;
- QoL instruments are not enough sensitive to detect differences;
- sample of patients is underpowered;
- period of treatment is too short to reveal long term changes.

Therefore more appropriate instruments to evaluate QoL of PKU patients seems to be required.

WHOQOL-100 [20] and PedsQL are two questionnaires recently devised to better evaluate QoL as compared to previous instruments.

In view of the above, the present study aimed to assess WHOQOL-100 and PedsQL questionnaires to score personal and parental perception of QoL in a sample of PKU patients affected by mild and classical PKU. Indeed, QoL of patients with mild and classical PKU have never been compared using up-to-date instruments thus far.

## Methods

### Study planning and sample portrayal

This study was conducted from March 2012 till July 2012, as a result of the collaboration of Padua University Hospital (enrolling pediatric patients) with the Neurological Unit of St. Bassiano Hospital in Bassano del Grappa (enrolling adult patients). The study protocol followed the principles of the Declaration of Helsinki and ICH/GCP and was approved by the Ethical committee of the Padua University Hospital. All recruited patients and/or their respective parents signed an informed consent.

A total of 30 mild BH_4_ potentially respondent patients have been selected to participate to the study. 3 of them were excluded due to age criteria (patients under 4 years of age), 2 were excluded due to the presence of mental impairment and 3 did not give their consent to participate to the study. An almost identical number of classical PKU patients were then recruited considering gender, disease severity and age-matching criteria.

Thus 22 BH_4_ responsive patients affected by mild PKU and 21 classical PKU patients treated with diet were recruited in this study. Information on age (in years), gender, treatment type (BH_4_ vs. diet), treatment length (months of current treatment, diet or BH_4_ therapy), employment status of adult patients (student, employed, unemployed) and educational level of pediatric (nursery, primary school, junior secondary school) or adult patients (secondary school or post-graduate education) were collected.

Patients affected by mild PKU treated with BH_4_ were included if:

- PKU was diagnosed by neonatal screening;
- Phe blood level ranged between 600 and 1200 μmol/L;
- a significant decrease of blood Phe level (>30%) following a BH_4_ loading test (performed with a BH_4_ dosage of 20 mg/kg/day).

Genetic analysis was conducted in all patients (both in mild PKU patients treated with BH_4_ as well as in classical PKU patients on diet). All patients with mild PKU were, thereafter, treated with a BH_4_ dosage of 10 mg/Kg/day, as suggested by Kure et al. [11], for a time period of 1 to 11 years. Until 2009 BH_4_ was provided by Schircks Laboratories in Switzerland; subsequently there was a switch to sapropterin dihydrochloride (Kuvan®, Merck Serono, Germany). All patients on BH_4_/sapropterin treatment were allowed a relevant relaxation of the dietary restriction [21].

Analysis of Phe was performed using liquid chromatography/tandem mass spectrometry (CC-MS/MS) as previously described [22].

### Technical instruments and questionnaires

A general instrument for QoL perception was administered to all study subjects.

In detail, for pediatric patients (6-16 years old) the Pediatric Quality of Life Inventory, PedsQL, was employed, an instrument measuring several aspects of QoL including a child self-report and a parent proxy-report format [23].

PedsQL consists of Likert scale items and yields a score scale for Physical Functioning, Emotional Functioning, School Functioning and Social Functioning. Items are reverse-scored and linearly transformed in a 0-100 scale, with higher scores corresponding to higher QoL. The PedsQL was self-administered for children 8-16 years old and by parents and administered by an interviewer to children 6-7 years old. In order to analyse the participants’ scores (children and their respective parents), results were compared with age-matched normative data controls taken from literature [24]. The Test of Anxiety and Depression in Infancy and Adolescence, TAD, was also used, an inventory including a self and a proxy-report [25]. This instrument enables to investigate the symptoms associated with anxiety and depression.

For adult patients (18-35 years old) the World Health Organization QoL score (WHOQOL-100) was employed, recording the global perception of their own QoL [20]. Items are linearly transformed into a 4-20 scale, with higher scores corresponding to higher QoL. This instrument was previously used also to test QoL in patients with other inherited metabolic diseases [8]. Age-matched normative data (age range: 18-44 years) taken from literature [26] was considered in the analysis.

The Beck Depression Inventory, BDI, a self-report instrument, which is both clinical and diagnostic, was also used to evaluate the presence and the degree of depression [27]. In this instrument the total score is compared to a reference value, to determine the severity of depression. Higher total scores are associated with more severe depressive symptoms.

Finally, the State-Trait Anxiety Inventory form Y (STAI-Y) was employed, an instrument which investigates the level of anxiety in adult patients. This inventory discriminate between state anxiety, for instance a transitory emotional condition, from trait anxiety, defined as a personality trait [28].

### Statistical analysis

The ANOVA test was employed to compare the mean values of Phe of BH_4_ patients and patients on diet. Four univariable linear regression models were employed to assess the association between QoL and Phe levels at assessment day and over the past 12 months in both groups (mild PKU on BH_4_ treatment and classical patients on diet). Two separate multivariable linear regression models were set up to investigate the effect of various predictors on QoL (as continuous outcome) perceived by the patients. The first model was fitted only on pediatric PKU patients controlling for age, sex, treatment type (BH_4_ vs. diet), length of current treatment (diet or BH_4_) and educational level. The second model was fitted only on adults PKU patients, controlling for age, sex, treatment type (BH_4_ vs. diet), length of current treatment (diet or BH_4_), educational level and employment status. Results of all above models were expressed as regression coefficients (RC) with 95% confidence interval (95% CI).

The analysis was conducted with Stata 13 statistical package (Stata Corporation, College Station, Texas, USA).

## Results

### Patient sample

Table 1 shows the distribution of the 43 PKU patients (20 females and 23 males) aged between 6 and 35 (mean 17.1 ± 9.0): 22 BH_4_ responsive patients (aged between 6 and 35 years; mean = 15.4 ± 9.5) affected by mild PKU, and 21 classical PKU patients treated with diet (aged between 8 and 34 years; mean = 18.9 ± 8.3).

**Table 1:** **Frequency distribution of the 43 PKU patients**

Variables	Strata	No. (%)	Mean ± SD	Mean ± SD normative population (Child Self-report)	Mean ± SD normative population (Parent proxy-report)
Gender	Female	20 (46.5)			
	Male	23 (53.5)			
Age (years)			17.09 ± 8.98		
Patients	Adult	17 (40.0)			
	Pediatric	26 (60.0)			
Education level (pediatric patients)	Nursery	3 (11.5)			
	Primary school	12 (46.2)			
	Junior secondary school	11 (42.3)			
Education level (adult patients)	Secondary school	14 (82.4)			
	Post-graduate	3 (17.6)			
Employment status (adult patients)	Student	5 (29.4)			
	Employed	9 (52.9)			
	Unemployed	3 (17.7)			
Treatment type	Diet	21 (48.8)			
	BH_4_	22 (51.2)			
Treatment length (months)			209.65 ± 140.02		
QoL pediatric (range: 0-100)			79.26 ± 82.97	79,37 ± 10 [24]	81,74 ± 11,21 [24]
QoL adults (range: 4-20)			16.47 ± 0.51	14,9 ± 2,9 [26]	

Number (No.), percentage (%), mean and standard deviation (SD).

Patients were broken down as follows:

- a group of 26 pediatric patients aged between 6 and 16 years with their respective parents;
- a second group of 17 adult patients aged between 17 and 35 years.

Three pediatric patients (11.5%) were attending nursery, twelve (46.2%) attended primary school and eleven (42.3%) attended junior secondary school. Fourteen adults patients (82.4%) had a secondary school education and three (17.6%) had a post-graduate education. Five adult patients (29.4%) were students, nine (52.9%) were employed and three (17.7%) were unemployed.

The average length of current treatment (diet or BH_4_) was 209.65 ± 140.02 months. The mean QoL of pediatric patients was 79.26 ± 82.97, whereas in adults it was 16.47 ± 0.51.

Tables 2 and 3 presents the biochemical, therapeutic and personal data of all patients:BH_4_ was generally well tolerated and no adverse events were reported over the years.

- 21 classical PKU patients treated with diet alone;
- 6 adult patients treated with sapropterin alone;
- 8 patients (all pediatric patients) treated with sapropterin as well as low protein foods and natural proteins (mainly from animal sources);
- 8 patients (7 children and 1 adult) requiring a combined treatment of sapropterin along with Phe restricted diet and a supplementation of Phe-free amino acid compounds.

**Table 2:** **Distribution of pediatric patients: sex, age, Phe levels**

Pediatric patients				
Pediatric patients on BH _4_ treatment*	Age	Sex	Phe values ^a^ on the day of assessment	Mean Phe values ^a^ over the course of the previous 12 months
**I**	6	F	313	292
**II**	10	M	416	361
**III**	8	F	358	350
**IV**	8	F	326	250
**V**	10	M	373	254
**VI**	10	F	664	553
**VII**	7	M	268	261
**VIII**	16	F	189	256
**IX**	6	M	254	335
**X**	10	F	222	224
**XI**	6	M	142	166
**XII**	11	F	240	251
**XIII**	16	F	309	364
**XIV**	16	M	251	268
**XV**	7	F	259	271
**Pediatric patients on dietary treatment**	**Age**	**Sex**	**Phe values** ^**a**^ **on the day of assessment**	**Mean Phe values** ^**a**^ **over the course of the previous 12 months**
**XVI**	15	M	471	318
**XVII**	8	M	473	343
**XVIII**	15	F	611	394
**IXX**	15	M	262	386
**XX**	8	M	273	291
**XXI**	10	F	396	320
**XXII**	13	F	128	476
**XXIII**	13	F	239	216
**XXIV**	9	F	288	321
**XXV**	16	F	563	678
**XXVI**	12	M	254	206

*10 mg/Kg/day for each patient.

^a^Expressed in μmol/L.

**Table 3:** **Distribution of adult patients: sex, age, Phe levels**

Adult patients				
Adults patients on BH _4_ treatment*	Age	Sex	Phe values ^a^ on the day of assessment	Mean Phe Values ^a^ over the course of the previous 12 months
**I**	26	M	273	255
**II**	35	M	502	541
**III**	30	M	654	586
**IV**	32	F	683	646
**V**	21	F	652	666
**VI**	18	M	349	414
**VII**	30	M	236	195
**Adults patients on dietary treatment**	**Age**	**Sex**	**Phe values** ^**a**^ **on the day of assessment**	**Mean Phe values** ^**a**^ **over the course of the previous 12 months**
**VIII**	28	F	922	1032
**IX**	27	F	624	608
**X**	18	F	635	557
**XI**	28	M	1359	1238
**XII**	27	M	917	784
**XIII**	17	F	481	442
**XIV**	30	M	1120	950
**XV**	29	M	735	638
**XVI**	34	M	782	800
**XVII**	24	F	682	719

*10 mg/Kg/day for each patient.

^a^Expressed in μmol/L.

Genetic analysis showed previously described BH_4_ responsive variants [15, 19, 29–35] of PAH gene and previously known classical PKU variants [36].

### Blood Phe levels

Blood Phe concentrations of all patients on BH_4_ treatment were within the therapeutic range both on the day of assessment and over the course of previous 12 months, in compliance with the Italian guidelines (0-12 years range: 120-360 μmol/L; >13 years range: 120-600 μmol/L) [13]. Phe tolerance increased 2-4 times above normal values following BH_4_ treatment [21].

Tables 2 and 3 shows that the mean blood Phe concentration in pediatric patients on diet regimen fell within the borderline range both on the day of assessment (mean = 357 ± 150 μmol/L) and over the course of previous 12 months (mean = 359 ± 131 μmol/L).

The mean blood Phe concentration in adult patients on diet regimen was above the recommended upper limit both on the day of assessment (mean = 826 ± 261 μmol/L) and over the course of previous 12 months (mean = 777 ± 240 μmol/L).

The mean Phe levels of mild PKU patients on BH_4_ were significantly lower than classical PKU patients on diet (Table 4).

**Table 4:** **ANOVA test comparing the mean Phe level of patients with classical PKU on diet and mild PKU on BH**_**4**_

	Mean ± SD	RC (95% CI)	p value	
**Classical PKU patients on diet**	579.95 **±** 315.75	*Reference*	<0.01	
**Mild PKU patients on BH**_**4**_	362.09 **±** 164.36	-217.86 (-371.85; -63.87)		

Mean, standard deviation (SD), 95% confidence interval (95%CI) and p value.

Finally, QoL of both groups (mild PKU patients on BH4 and classical PKU patients on diet) was significantly correlated with the average value of Phe over the previous 12 months and Phe value measured on the day of assessment (see Table 5).

**Table 5:** **Association between Phe levels (in the previous 12 months and at assessment) and QoL scores in mild PKU patients on BH**_**4**_ **treatment and classical patients on diet expressed as regression coefficients (RC) with 95% confidence intervals (95%CI) and p values**

Mild PKU patients on BH _4_			
	RC	95% CI	p value
phenylalanine in previous 12 months	- 0.13	(-0.21; -0.05)	< 0.01
phenylalanine at assessment	- 0,11	(-0.18; -0.04)	< 0.01
**Classical PKU patients on diet**			
	**RC**	**95% CI**	**p value**
phenylalanine in previous 12 months	- 0.08	(-0.12; -0.04)	< 0.01
phenylalanine at assessment	- 0.08	(-0.11; -0.04)	< 0.01

### Quality of life

Parents’ perception of patients QoL showed no significant mean differences as compared to normative children’s data. Moreover, patients’ perception of QoL did not significantly differ from normative data. There was no statistically significant correlation between parents and children perception of QoL. The trend tended towards 0 in every instrument observed (Correlation Coefficient = 0.148, p > 0.05).

As it can be seen from Table 6, after holding constant the effects of all other predictors, mean QoL score in pediatric patients was significantly higher in pediatric patients with mild PKU on BH_4_ treatment (RC = 15.12; 95% CI: 3.08; 27.15) as compared to pediatric patients affected by classical disease treated with diet (reference). A significantly increase of QoL (RC = 0.11; 95% CI: 0.01;0.21) was also observed in long treated patients (either diet or BH_4_).

**Table 6:** **Multivariable linear regression model for the quality of life in the 26 pediatric PKU patients**

Variables	No (%)	RC (95% CI)	p value
Gender			
Female	12 (46.2)	*Reference*	0.66
Male	14 (53.8)	- 1.59 (-8.91; 5.74)	
Age (years)			
10.81 ± 3.51		- 0.33 (- 2.96; 2.29)	0.79
Education level			
Nursery	3 (11.54)	*Reference*	0.80
Primary school	12 (46.15)	- 1.79 (- 15.88; 12.31)	
Junior secondary school	11 (42.31)	5.96 (-20.07; 31.98)	
Treatment			
Diet	11 (42.3)	*Reference*	0.02
BH4	15 (57.7)	15.12 (3.08; 27.15)	
Length of current treatment (months)			
89.08 ± 61.86		0.11 (0.01; 0.21)	0.03

Number (No.), percentage (%), regression coefficients (RC) with 95% confidence intervals (95%CI) and p values. Model fitted on 26 complete observations.

Similar results were also observed for adult patients (Table 7). By holding constant the effect of other terms, mean QoL scores were indeed significantly higher in adult patients with mild PKU under BH_4_ treatment (RC = 7.89; 95% CI: 2.47; 13.31) as compared to patients with classical PKU on diet (reference). In adult patients QoL was also significantly lower in males (RC = -2.58; 95% CI: -4.44; -0.72) as compared to females (reference) but was higher in those with postgraduate education (RC = 3.26; 95%CI: 1.33; 5.18) as compared to patients with secondary school education (reference). Lastly, adult patients, employed (RC = - 2.22; 95% CI: -4.26; -0.18) or unemployed (RC = - 2.73; 95% CI: -4.79; -0.66), had a significantly lower average QoL scores as compared to students (reference).

**Table 7:** **Multivariable linear regression model for the quality of life in the 17 adult patients**

Variables	No. (%)	RC (95% CI)	p value
Gender			
Female	8 (47.1)	*Reference*	0.01
Male	9 (52.9)	-2.58 (-4.44; -0.72)	
Age (years)			
26.71 ± 5.46		-0.16 (- 0.33; 0.02)	0.08
Education level			
Secondary School	14 (82.4)	*Reference*	<0.01
Post-graduate	3 (17.6)	3.26 (1.33; 5.18)	
Employment status			
Student	5 (29.4)	*Reference*	0.01
Employed	9 (52.9)	- 2.22 (-4.26; -0.18)	
Unemployed	3 (17.7)	- 2.73 (-4.79; -0.66)	
Treatment			
Diet	10 (58.8)	*Reference*	0.01
BH_4_	7 (41.2)	7.89 (2.47; 13.31)	
Length of current treatment (months)			
209.65 ± 140.02		0.03 (0.01; 0.21)	0.01

Number (No.), percentage (%), regression coefficients (RC) with 95% confidence intervals (95%CI) and p values. Model fitted on 17 complete observations.

### TAD, BDI and STAI-Y

TAD, BDI and STAI-Y scores were within normal values in all patients and none of the estimates reached statistical significance. In particular at the TAD test:

- 2 parents out of 15 reported a mild level of depression in their children;
- 3 parents out of 15 reported a mild level of anxiety in their children;
- 2 parents out of 15 reported a mild level of social difficulties in their children;
- only 1 pediatric patient under BH_4_ treatment reported mild level of anxiety.

In the adult population, the BDI did not detect any pattern of depression whereas the STAI-Y displayed that two adults on dietary treatment reported anxiety (data not shown in any table).

## Discussion

In the present study QoL was evaluated among 22 patients affected by mild PKU treated with BH_4_ and 21 patients with classical PKU on diet. Although QoL global scores were normal in both groups, both pediatric and adult patients affected by mild PKU treated with BH_4_ reported a significantly higher QoL scores than patients with classical PKU on diet. Nevertheless, both in the pediatric and adult groups a significant increase of QoL was observed in long treated patients, irrespective if it was diet or BH_4_ therapy. This finding seems to suggest that both treatments (diet or BH_4_) are effective to improve QoL of PKU patients in the long run. This could be attributable to increasing compliance to treatment over time (irrespective of treatment type).

Diet remains the mainstay for patients with classical disease, whereas those affected by mild PKU and struggling to adhere to diet regimen have the opportunity to rely on BH_4_ medical treatment.

Several studies support the effectiveness of BH_4_ in lowering blood Phe levels. However, there is lack of data for long term treated patients and the biochemical effect still does not have a clear counterpart in terms of QoL and behaviour for PKU patients on diet [15, 16], whose QoL scores were reported to be similar to healthy controls [17, 37, 38]. Globally speaking, all previous studies suggested that PKU patients have QoL scores similar to the healthy populations. Only Cotugno et al. [39] reported lower parental QoL scores as compared to non-PKU patients. Gentile et al. [40] recently stated that this could be due to a sort of “hidden disability” effect, a cumulative impact of relatively subtle symptoms not easy to be detected by currently used QoL questionnaires. Nevertheless, adolescents and adult patients reported that adherence to diet is arduous [4–6, 41] and diet treatment significantly limits their daily life activities.

In this study WHOQOL-100 and PedsQL were employed, two recently developed inventories to assess global QoL. In particular, WHOQOL-100 was developed to further improve QoL assessment in comparison to previous instruments, both by extending the number of the questions and by enabling cross-cultural comparisons. Previous instruments were developed based upon SF-36, a health related QoL scale encompassing objective measures in addition to perceived state [42]. Above all, in the SF-36 instrument physical health subscales refers largely to physical symptoms (such as pain, mobility, fatigue) and is probably more adequate to investigate QoL in other medical conditions. In disorders such as PKU, where physical illness is less severe than daily life limitations, other aspects of QoL may be more relevant to evaluate. Moreover, WHOQOL-100 has a good discriminatory power in its physical and independence domains [43] and includes a detailed section, under the independence domain, focused on the influence of medication and medical aids in daily life activities. In this sense, WHOQOL-100 encompasses a larger number of dimensions (level of independence, personal relationship, positive feelings, sexual activity and also dependence on medical substances/aids) that seems more suitable to better assess PKU treatment constraints.

However, in the present study parents’ perception of patients’ QoL showed no significant mean differences as compared to parents’ of healthy children. Moreover, patients’ perception of QoL did not significantly differ from normative data. Even advanced and detailed QoL instruments, as those employed in the present study, therefore are still not able to detect some criticalities in the assessment of QoL for PKU patients. Future inventories should be improved by focusing more on the perception of personal limitations and on the social implications associated with a chronic and restrictive dietary regimen.

Male gender was found to be significantly associated with lower QoL among adult patients. This evidence was never reported before in any study and deserves attention. The latter finding, not confirmed for male pediatric patients, might be explained by parental control. In this study adult students had a significantly higher QoL score than employed and unemployed patients. The impact of education and socio-economic status is indeed confirmed by the significantly higher QoL score found in adult patients with higher education in this study.

The chronicity and complexity of the treatment makes it hard to manage PKU both for patients and parents, often engendering anxiety and fear of not being able to adequately control the course of the disease [18]. Also, parental mental reaction certainly has an impact on children’s emotional development [44, 45]. In addition, high levels of stress are related to the burden of treatment management, with a subsequent increased risk of depression and anxiety [9, 46]. Indeed internalizing psychiatric disorders such as anxiety and depression have been frequently described in association with PKU [46]. In the present study BDI, TAD and STAI-Y were also administered, questionnaires never previously used to evaluate PKU patients. In terms of psychiatric symptoms, no patients scored for depression. By contrast, one patient reported mild levels of anxiety.

Several previous studies have focused on behavioural, psychiatric and social functioning in PKU patients [9, 40, 46] with controversial results [47–49]. Although it is not possible to depict a clear psychological phenotype, internalizing traits (such as depressed mood, phobias and anxiety) have been frequently described, mostly in adult women. The results of this study confirmed that some PKU patients presented internalizing personality traits (predominantly anxiety maladaption) hard to define by currently used psychiatric inventories.

From a biochemical perspective, mild PKU patients on BH_4_ treatment showed better metabolic control, with lower mean blood Phe levels and lower variability towards mean values (lower SD). When compared with QoL scores, Phe level was significantly correlated with QoL in both groups, at assessment as well as over the course of previous 12 months.

### Limitations

The results of the present study should be interpreted with caution.

Although the distribution by gender (21 males vs. 22 females) and disease severity (22 mild vs. 21 classical) was fairly homogeneous, the age distribution of both groups (patients with mild PKU on BH_4_ and patients with classical PKU on diet) was heterogeneous.

Moreover, better QoL scores might be due to a less severe disease and a better self-assessment. On the other hand it can be argued that global QoL scores may be simply lower in patients with classical PKU as compared to those affected by mild disease, due to the burden of diet. In the present study mild PKU patients on BH_4_ indeed slightly differed from classical ones on diet as the former were affected by milder disease.

Despite a relatively small sample size, this work provided significant results which might contribute to assess and improve currently used inventories for the detection of QoL in PKU patients. Some statistical estimates may become significant with a larger study. Probably a multi-center study would be required to enlarge the cohort and reach stronger statistical power.

Finally, since the results come from a cross sectional study, a stronger study design is recommended to confirm the above findings.

## Conclusions

The aim of the present study was to investigate various aspects of QoL in a group of patients affected by mild and classical PKU, in relation to different treatment regimen (BH_4_ vs. diet). Global QoL scores were found to be significantly higher in patients with mild PKU treated with BH_4_ as compared to patients with classical disease on dietary treatment, both in the adult and the pediatric group. Some specific categories of adult patients (males, those less educated and non students) reported a significantly lower QoL score and should be investigated more in depth, targeting categories patients with potential low treatment compliance. Finally, this study confirmed that currently used QoL inventories constitute a significant advance from instruments employed in the past; however further improvements of these inventories are still recommended.

## Abbreviations

PKU: Phenylketonuria
PAH: Phenylalanine hydroxylase
Phe: Phenylalanine
BH4: Tetrahydrobiopterin
QoL: Quality of Life
WHOQOL-100: Word Health Organization Quality Of Life questionnaire-100.
